# Role of PCSK9 in breast cancer: a systematic review of its mechanistic pathways and clinical relevance

**DOI:** 10.3389/fphar.2026.1849027

**Published:** 2026-08-13

**Authors:** Meidi Utami Puteri, Vicko Suswidiantoro, Rahma Nabila, Renata Setiawan, Nuriza Ulul Azmi, Fadlina Chany Saputri, Yukihide Watanabe, Mitsuyasu Kato

**Affiliations:** 1 Laboratory of Pharmacology and Toxicology, Faculty of Pharmacy, Universitas Indonesia, Depok, West Java, Indonesia; 2 National Metabolomics Collaborative Research Centre, Faculty of Pharmacy, Universitas Indonesia, Depok, West Java, Indonesia; 3 Faculty of Pharmacy, Universitas Indonesia, Depok, Indonesia; 4 Laboratory of Pharmacology and Toxicology, Pharmacy Department, Faculty of Health, Universitas Aisyah Pringsewu, Lampung, Indonesia; 5 Department of Experimental Pathology, Institute of Medicine, University of Tsukuba, Ibaraki, Japan

**Keywords:** breast cancer, cancer progression, clinical relevance, LDLR, pcsk9

## Abstract

**Introduction:**

Breast cancer is a heterogeneous disease in which accurate classification based on biomarker expression is essential for effective therapeutic decision-making. Consequently, the identification of novel biomarkers remains a research priority. Proprotein convertase subtilisin/kexin type 9 (PCSK9), a key regulator of low-density lipoprotein (LDL) metabolism *via* its interaction with the LDL receptor (LDLR), has been extensively studied in cardiovascular disease. More recently, emerging evidence indicates that PCSK9 is highly expressed in several malignancies and may contribute to cancer progression, including in breast cancer.

**Methods:**

A systematic literature review was conducted in accordance with Preferred Reporting Items for Systematic Reviews and Meta- Analyses (PRISMA) guidelines. PubMed (NCBI), Scopus, and Google Scholar were searched for studies published between 9 and 16 September 2025. Eligible studies included experimental (*in vitro, in vivo*, and *in silico*) and clinical investigations evaluating the role of PCSK9 in breast cancer. Given the heterogeneity in study designs and outcomes, findings were synthesized narratively.

**Results:**

Nineteen studies met the inclusion criteria. Preclinical evidence consistently demonstrates that PCSK9 promotes tumorigenesis by enhancing cell proliferation, migration, invasion, and metastasis, supporting its potential as a biomarker and therapeutic target. However, clinical findings remain inconsistent, with conflicting evidence regarding the association between PCSK9 expression and patient prognosis.

**Conclusion:**

In conclusion, while preclinical studies indicate a pro-tumorigenic role for PCSK9, its clinical significance in breast cancer appears to be context-dependent. The effects of PCSK9 vary according to molecular subtype and are influenced by the tumor microenvironment. Further well-designed studies are needed to clarify its prognostic value and potential as a target for personalized therapy.

## 1 Introduction

### 1.1 Background

Despite advances in early detection and a range of treatment options—from surgery and radiation to chemotherapy and targeted therapy—breast cancer remains the most frequently diagnosed cancer in women and a leading cause of cancer-related deaths worldwide (Arnold et al., 2022). The biggest challenge lies in the nature of the disease itself: it is highly complex, prone to recurrence, and spreads easily (Zafar et al., 2025). These diverse biological characteristics are categorized into several molecular subtypes, such as Luminal A/B, human epidermal growth factor receptor 2 (HER2)-positive, and triple-negative breast cancer (TNBC) (Hashmi et al., 2023), This reality drives active research into new biological markers (biomarkers) and treatment targets. The goal is clear: to be able to predict the progression of the disease more accurately and develop more personalized and targeted treatments for each patient.

Recent studies increasingly highlight the crucial role of lipid metabolism dysregulation in the pathogenesis of breast cancer (Huang et al., 2025). Lipids serve not only as essential structural components of cell membranes but also as versatile signaling mediators that influence cell growth, apoptosis, and inflammation (Xiao et al., 2024). Among the lipid-regulating pathways, proprotein convertase subtilisin/kexin type 9 (PCSK9) has attracted growing attention as a molecular bridge linking lipid homeostasis with cancer biology (Bhattacharya et al., 2021).

The expansion of PCSK9 research from a traditional focus on lipid metabolism to more complex roles in oncology is supported by recent bibliometric trends (Luo et al., 2023). While early studies primarily focused on the mechanisms of low-density lipoprotein (LDL) reduction, the field has recently entered a new era marked by an explosion of research on PCSK9’s role in inflammation and cancer (Bao et al., 2024; Poirier et al., 2008; Luo et al., 2023). A pioneering study by Liu et al. (2020) reported that PCSK9 inhibition—whether by genetic deletion or antibody treatment—enhances anti-tumor immunity by increasing MHC I surface expression and promoting cytotoxic T-cell infiltration, thereby improving the efficacy of immune checkpoint therapy (Liu et al., 2020). Strengthening the finding, we have summarized the molecular mechanisms by which PCSK9 drives cancer progression, including its roles in cell proliferation, programmed cell death, immune modulation, and metastasis (Azmi et al., 2025). Thus, exploring the roles of PCSK9 beyond the cardiovascular domain may deepen the understanding of tumor biology and open promising opportunities for therapeutic intervention in breast cancer.

### 1.2 Breast cancer

Modern research classifies breast cancer into several molecular subtypes—such as Luminal A/B, HER2-positive, and TNBC—a scheme that reflects its vast genetic and clinical diversity. This classification is based on the status of hormone receptors (estrogen, progesterone) and HER2, with each category exhibiting unique biological characteristics and responses to treatment (Hashmi et al., 2023; Nolan et al., 2023).

Another important discovery is disruption in fat (lipid) metabolism, now recognized as a major driver of the disease progression. To fuel their aggressive growth, cancer cells rewire their metabolism to boost the synthesis and uptake of lipids (Xiao et al., 2024). These fats then serve as both an energy source and as building blocks for cellular expansion (Wan et al., 2025).

In this process, cholesterol plays a dual role. On one hand, it regulates cell membrane fluidity and supports cell signalling through “lipid rafts” (PI3K/AKT/mTOR and MAPK/ERK) which ultimately activates cancer-driving pathways (Paukner et al., 2022; Xiao et al., 2024). Additionally, cholesterol metabolites can interact with estrogen receptors and amplify their signaling, directly fueling the growth of hormone-dependent cancers (Fernández-Suárez et al., 2021; Sant et al., 2025). Collectively, these insights emphasize the significance of molecules that maintain cholesterol homeostasis, particularly PCSK9, as key regulators of tumor growth and patient outcomes.

### 1.3 Overview of PCSK9

PCSK9, originally termed neural apoptosis-regulated convertase 1 (NARC-1), was subsequently identified as a unique member of the proprotein convertase enzyme family (Seidah et al., 2003). NARC-1 was originally identified as a molecule that promotes neuronal survival and influencing synaptic remodeling, implying its key involvement in both brain development and neurodegenerative processes (Poirier et al., 2006).

PCSK9 is a secreted serine protease that is predominantly produced in the liver but is also expressed in several extrahepatic organs, including the intestine, kidney, and central nervous system. Initially, PCSK9 was identified as a crucial regulator of plasma low-density lipoprotein cholesterol (LDL-C) through its interaction with the LDL-receptor (LDLR) (Bao et al., 2024; Zheng et al., 2025). By binding to LDLR on the surface of hepatocytes, PCSK9 directs the receptor toward lysosomal degradation rather than allowing it to return to the cell membrane, thereby reducing the clearance of LDL-C from the bloodstream (Zheng et al., 2025).

Beyond its role in fat processing, the protein PCSK9 also actively fuels cancer growth through a few key methods (Ungvari et al., 2025a). It helps control cholesterol levels, which cancer cells greedily consume as a building block for their membranes and internal signals. Perhaps more cunningly, PCSK9 can act as a kind of disguise for the tumor; by breaking down “identification tags” on the cell surface, it makes cancer cells harder for the immune system to find and destroy (Ungvari et al., 2025a; Wang W. et al., 2024). Moreover, PCSK9 has been shown to switch on specific growth signals inside the cell, such as the MAPK and PI3K/AKT pathways, which encourage tumor expansion and help the cells resist self-destruction (Fu et al., 2022).

Recent studies have reported upregulated PCSK9 expression in several types of cancers, such as breast, liver, and colorectal cancers (Cui et al., 2025). Higher PCSK9 levels are frequently associated with more aggressive tumor phenotypes and poorer clinical outcomes (Ungvari et al., 2025a; Wang Y. et al., 2024). Altogether, these findings suggest that PCSK9 may serve not only as a biomarker for cancer progression but also as a promising therapeutic target, either alone or in combination with immune checkpoint inhibitors or agents targeting lipid metabolism.

### 1.4 Problem statement

Breast cancer is still a major global health concern. Effective treatments of breast cancer depend on precise biomarker analysis and expression, which are essential for the development of targeted therapies. One promising biomarker for targeted breast cancer therapy is PCSK9. To note, PCSK9 has been extensively studied in relation to cardiovascular diseases; several inhibitors, such as monoclonal antibodies and siRNA, have been developed. Therefore, applying this knowledge to breast cancer research could expedite the development of a PCSK9 inhibitor for cancer treatment. Although, preclinical experiments show consistent and promising results for targeting PCSK9 in breast cancer, some clinical studies yield conflicting or mixed results. This highlights the need for extensive and comprehensive literature studies on the role of PCSK9 in breast cancer.

### 1.5 Research question

Are the mechanistic pathway and clinical relevance of PCSK9 in breast cancer positively connected?

### 1.6 Aims and objectives

- To provide an overview of the roles of PCSK9 in breast cancer, from its molecular mechanism to clinical studies.
- To confirm the experimental and clinical data, and whether they are positively linked.
- To determine the roles of PCSK9 in breast cancer.
- To analyze the potential and limitations of targeting PCSK9 in breast cancer.

## 2 Research methodology

### 2.1 Study design

This study used the Preferred Reporting Items for Systematic Reviews and Meta- Analyses (PRISMA) guidelines to guide this study. It is a systematic narrative literature review providing the recent literature without the requirement of ethical approval. Moreover, because of the variations in methodologies, populations, and outcome measures across the included studies, a meta-analysis was not done. Findings were categorized and analyzed according to two study types: experimental and clinical studies. Key results from each investigation were systematically extracted and compiled into evidence tables to provide a comparison across studies. The synthesis aimed to explore mechanistic pathways through *in silico, in vitro*, and *in vivo* studies, as well as their clinical relevance based on genomic and patient databases analysis, to evaluate key findings among different research approaches and provide a comprehensive assessment of the role of PCSK9 in breast cancer.

### 2.2 Search strategy

To ensure reproducibility, a systematic literature search was conducted from September 9th to 16th, 2025, with regular updates performed until manuscript submission. A comprehensive search strategy was applied across three primary databases: PubMed (*via* NCBI), Scopus, and Google Scholar. The search combined controlled vocabulary (such as MeSH terms) and free-text keywords using Boolean operators: (“PCSK9” OR “Proprotein Convertase Subtilisin/Kexin Type 9”) AND (“Breast Cancer” OR “Mammary Carcinoma” OR “Breast Tumor” OR “Breast Neoplasm”). We included all peer-reviewed, English-language studies examining the role of PCSK9 in breast cancer across experimental (*in vitro* and *in vivo*), genetic (Mendelian randomization), and clinical approaches. Literature types such as theses, dissertations, and conference proceedings were excluded to maintain a focus on primary, peer-reviewed data.

### 2.3 Data extraction

Three reviewers (Meidi Utami Puteri [MUP], Rahma Nabila [RN], and Renata Setiawan [RS]) independently screened the studies. Each reviewer assessed eligibility by voting ‘yes’ (eligible), ‘no’ (ineligible), or ‘maybe’ (uncertain). Studies receiving two affirmative votes (‘yes’ or ‘maybe’) were advanced to full-text review, while those receiving two negative votes were excluded and categorized as ‘Irrelevant.’ Any discrepancies between reviewers were discussed and resolved through consensus. Following this, the reviewers assessed the full-text articles based on the predefined inclusion and exclusion criteria (Supplementary Table S1, Supplementary Material).

### 2.4 PRISMA flow diagram

**FIGURE 1:**
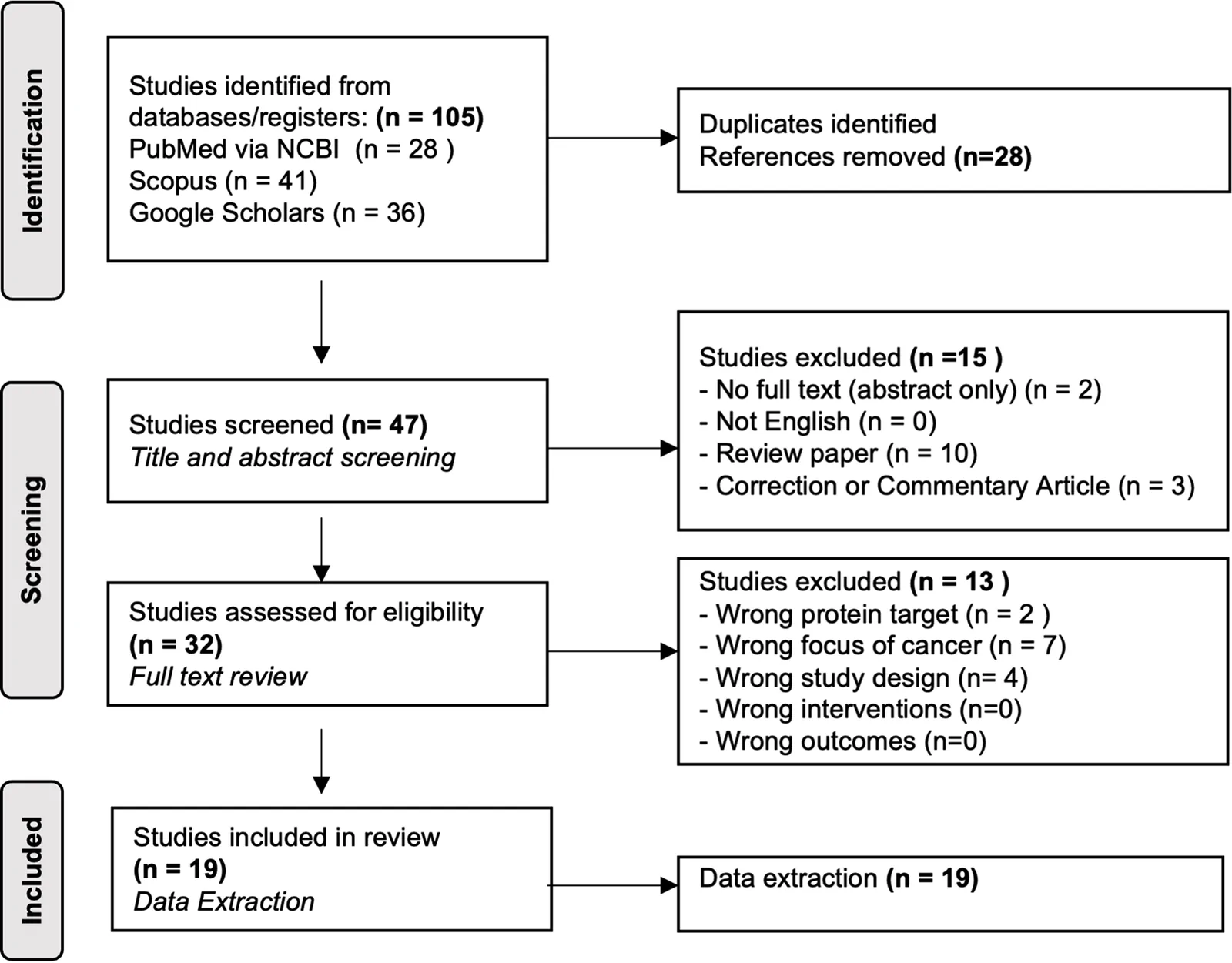
PRISMA diagram.

The PRISMA flow diagram (Figure 1) illustrates the overall selection process undertaken in this systematic review, depicting the number of records identified, screened, excluded, and ultimately included across each stage of the analysis. This visual summary ensures transparency in the study selection process and provides a clear overview of how the final pool of eligible studies was determined. To maintain consistency and accuracy in data handling, a standardized extraction template was developed. This form was used to systematically collect essential details from each full-text article, including general study information, sample characteristics, methodological approaches, and primary outcomes, as summarized in (Supplementary Table S1, Supplementary Material). Each study was independently reviewed by MUP, who performed the initial data extraction. The extracted data were subsequently verified by RS and RN through cross-checking against the original full-text publications to ensure precision and completeness. Following validation, the compiled data were synthesized to identify emerging patterns and key mechanistic insights relevant to the role of PCSK9 in breast cancer. Finally, a narrative synthesis was employed to integrate the findings and highlight their mechanistic and clinical significance across the different study types. Figure 1 shows the PRISMA flow chart.

### 2.5 Risk of bias assessment

Risk of bias was assessed according to study design. Animal studies were evaluated using the SYRCLE risk-of-bias tool, while *in vitro* studies were assessed using a modified methodological quality assessment framework. Mendelian randomization studies were evaluated based on the core MR assumptions, and non-MR clinical studies were assessed using the Quality in Prognosis Studies (QUIPS) tool (Yu et al., 2026; Bezemer et al., 2021).

## 3 Results

### 3.1 Study selection

We identified 105 articles using our search strategy. We eliminated duplicates, leaving 47 papers for title and abstract reviews. Following the initial screening, 32 articles met the requirements for full-text evaluation. Following the full-text screening, the review identified 19 papers suitable for inclusion. Of these, two articles focused on a protein target other than PCSK9, seven were not focused on breast cancer, and four used different study approaches. Table 1 depicts the inclusion and exclusion criteria for article selection.

**TABLE 1:** Inclusion and exclusion criteria.

Criteria	Inclusion	Exclusion
Date	The articles were published from 2015 to 2025 based on keywords.	Articles published before 2015
Language	English	Other than English
Type of Study	• Experimental studies: Approaches using mammalian cell cultures or animal models, ranging from bioinformatics-based screening to molecular biology experiments. • Clinical and epidemiological studies: Investigations involving human participants, patient-derived samples, or population data, including: o Clinical cohorts (hospital- or trial-based studies) o Observational studies (prospective, retrospective, or case-control) o Mendelian randomization studies (genetic-based causal inference analyses) o Analyses of public datasets (e.g., TCGA, GEO, GWAS) o Experimental validations using patient samples	• Review Article • Commentary Article • Irrelevant studies focusing on other topics than PCSK9 and breast cancer
Availability	Articles available as free full text or accessible through a university account	Articles with limited access (abstract only or no full text available

Risk of bias was assessed according to study design. Among the included animal studies, most domains were judged as having low risk of bias regarding baseline characteristics, incomplete outcome data, and selective reporting. However, several domains were classified as having unclear risk of bias due to insufficient reporting of randomization, allocation concealment, and blinding procedures. Among the *in vitro* studies, risk of bias was generally low to moderate. Most investigations employed well-characterized cell models and clearly reported experimental procedures and outcome measures. The primary concerns were related to limited replication across multiple experimental systems and the lack of external validation of mechanistic findings. Of the four Mendelian randomization (MR) studies, one was assessed as having low risk of bias, two as low–moderate risk, and one as moderate risk. The main concerns were population stratification and potential violations of the exclusion restriction assumption. Among the eight non-MR clinical studies, one was classified as low risk of bias, one as low–moderate risk, and six as moderate risk. The most common sources of bias were limited population representativeness and residual confounding. Detailed risk-of-bias assessments are presented in Supplementary Tables S2–S5.

### 3.2 Connection between breast cancer and PCSK9

Among the various risk factors for breast cancer, hyperlipidemia has attracted increasing attention, particularly in studies investigating the initiation of hormone-dependent breast cancer (Wei et al., 2021). Research indicates a direct relationship between elevated levels of LDL-C and very low-density lipoprotein cholesterol (VLDL-C) and the development of breast cancer (Nouri et al., 2022). A pioneering study from 1909 established a connection between cholesterol and cancer by observing “fatty nature” crystals in tumor samples (White, 1909). More recent research has highlighted the significance of the cholesterol biosynthetic pathway in the advancement of breast cancer, especially in cases of estrogen receptor-positive (ER+) breast cancer. Research also suggests a feedback loop between tumors and hyperlipidemia through a feed-forward mechanism that alters liver lipoprotein homeostasis, leading to increased production of LDL-C, which in turn supports tumor growth in various mouse models (Ehmsen et al., 2019).

Although the role of lipids—especially in ER-positive breast cancer—is well-known, establishing causal pathways has proven challenging. The role of the cholesterol pathway in cancer was once debated, however, data from The Cancer Genome Atlas (TCGA) have shown that genes controlling cholesterol synthesis are often overexpressed and play an important role in cancer development (Weinstein et al., 2013). Although clinical studies directly linking high cholesterol levels (hypercholesterolemia) to hormone-dependent breast cancer are still limited, growing evidence suggests that cholesterol has a significant influence on this type of cancer (Baek et al., 2017; Cedó et al., 2019; Mayengbam et al., 2021).

PCSK9 was initially recognized for its key role in cardiovascular diseases related to hypercholesterolemia by regulating LDL plasma levels (Testa et al., 2024). The connection between PCSK9 and LDL-C levels was identified through the study of a PCSK9 mutation in patients with familial hypercholesterolemia (Kent et al., 2017). It binds to hepatic LDLR and promote their degradation, which increases circulating LDL-C levels. Thus, we can emphasize that hyperlipidemia acts as a bridge linking the relationship between PCSK9 and breast cancer, particularly the hormone-dependent breast cancer (Cui et al., 2025). Notably, epidemiological research highlights that both cardiovascular diseases and breast cancer share modifiable risk factors and mechanisms, including hyperlipidemia.

Furthermore, growing evidence shows the relationship between PCSK9 not only in hormone-dependent breast cancer but also in other types of breast cancer (e.g., TNBC) and other cancers as well (Ruscica et al., 2024), suggesting the PCSK9 mechanism in promoting cancer does not depend solely on its role in LDLR. Indeed, in recent years, many studies have reported the pleiotropic role of PCSK9 in cardiovascular disease independently from its interaction with LDLR (Bao et al., 2024; Zheng et al., 2025). Reportedly,it also contribute to platelet activation by binding to Platelet CD36 (Qi et al., 2021) and to enhance inflammation through various mechanisms, including interactions with inflammatory receptors such as LOX-1 (Wang W. et al., 2024). Growing evidence also demonstrates how PCSK9 has a role not only in hormone-dependent breast cancer but also in TNBC (Li et al., 2025), in which disease initiation is unlikely to be related to lipid regulation. Given this context, thorough investigation of the mechanistic pathways by which PCSK9 may induce breast cancer progression, both dependent on and independent of LDLR. Additionally, incorporating clinical relevance studies will strengthen the studies and help determine the link between PCSK9 and breast cancer comprehensively. A comprehensive understanding of these mechanisms requires integration of molecular findings with clinically relevant evidence to clarify the mechanism by which PCSK9 contributes to tumor development and patient outcomes.

To address this, the present study systematically reviewed published papers and classified them into two main categories: experimental and clinical studies. Experimental studies utilized mammalian cell cultures or animal models, encompassing approaches that ranged from bioinformatics to molecular biology analysis. In contrast, the clinical studies are categorized as studies focused on human participants, patient-derived samples, or population-level data. This category included clinical cohorts (hospital- or trial-based studies), observational studies (prospective, retrospective, or case-control), Mendelian Randomization analyses (genetic-based causal inference), and examinations of public datasets such as the TCGA, *Gene Expression Omnibus* (GEO), and *Genome-Wide Association Study (*GWAS).

### 3.3 Experimental studies

Across the selected papers, we identified seven experimental studies that used mammalian cell cultures or animal models to study PCSK9 in breast cancer. Most of the studies aim to identify the molecular mechanism by which PCSK9 underlies breast cancer progression to make it as potential biomarker and targeted therapy. It is well established that PCSK9 regulates cholesterol by promoting the degradation of LDLR in lysosomes. However, within the tumor microenvironment, PCSK9 exhibits complex pleiotropic roles, To make it more clearly described, we classified them into LDLR-dependent mechanisms (Table 2) and LDLR-independent mechanisms (Table 3).

**TABLE 2:** Summary of experimental studies on PCSK9 and breast cancer with mechanisms involve LDLR.

Author (Year)	Population/Model	Intervention	Main findings
Momtazi-Borojeni et al. (2019b)	Female BALB/c mice bearing 4T1 mouse breast tumors cells inoculated subcutaneously.	Immunization with nanoliposomal anti-PCSK9 vaccine (L-IFPTA^+^); four doses subcutaneously at 2-week intervals. Groups: Control, Vaccine, Vaccine + Doxil, and Doxil alone	PCSK9 vaccination effectively inhibited PCSK9 and reduced tumor growth. • Anti-PCSK9 antibodies significantly decreased plasma PCSK9 levels. • Tumor growth inhibition: Vaccine 21%, Combination 48%, Doxil® 53% vs. control. • Time-to-endpoint extended (47 ± 10 days vs. 39 ± 9 days). • Modest survival gain (↑ 4.2%). → Suggests PCSK9 inhibition *via* vaccination suppresses breast cancer progression without adverse effects observed.
Abdelwahed et al. (2020)	*In vitro*: Human hormone-dependent breast cancer cell lines BT-474 (HER2 + /ER + ) and T47D (ER + ), and hepatic cell lines HepG2 and Huh-7 for PCSK9 secretion assay. *In vivo*: Athymic female nude mice implanted orthotopically with BT-474 cells in the mammary fat pad and fed a high-fat diet (HFD) to mimic hypercholesterolemia. Used also for post-surgical recurrence models.	Treatment with Pseurotin A (PS): • *In vitro*: 25–100 μM PS for 72 h to assess effects on PCSK9 and LDLR expression. • *In vivo*: 10 mg/kg PS administered orally daily. • Mechanistic assays: Molecular docking and Surface Plasmon Resonance (SPR) to confirm PS binding at the PCSK9–LDLR interface. • Biological assays: Western blot, IHC, and plasma measurements of cholesterol, 17β-estradiol, and CA15-3.	Dual inhibition mechanism of PCSK9: Pseurotin A suppresses both PCSK9 secretion (IC_50_ = 1.2 μM in HepG2) and PCSK9–LDLR binding, restoring LDLR levels. In hormone-dependent BC cells (BT-474, T47D), PS reduces PCSK9 expression and increases LDLR abundance. *In vivo*, PS (10 mg/kg, oral) significantly inhibits tumor growth and reduces local recurrence by 78.4% post-surgery. PS also decreases PCSK9 in tumors and liver, lowers total plasma cholesterol, and reduces CA15-3 recurrence marker by 77.4%. Overall, Pseurotin A acts as a first-in-class natural dual PCSK9-LDLR inhibitor that suppresses progression and recurrence of hormone-dependent breast cancer.
Liu (2024)	Female BALB/c mice (8 weeks old) bearing 4T1-Luc triple-negative breast cancer (TNBC) tumors. Mice were divided into normal diet and high-fat diet (HFD) groups to model hyperlipidemia; also included *in vitro* co-culture of TNBC (MDA-MB-231) and adipocyte (3T3-L1) cells.	*In vivo*: Mice were treated with polydatin (100 mg/kg intraperitoneally daily) under normal or high-fat diet conditions. *In vitro*: Co-culture assays (TNBC cells + adipocytes) with polydatin, PCSK9 siRNA knockdown, and PCSK9 overexpression were used to assess effects on lipid metabolism and PCSK9/LDLR regulation. Analyses included MTT, wound healing, transwell migration, ELISA, Western blot, qPCR, and IHC.	Polydatin significantly reduced TNBC proliferation, invasion, and lung metastasis in both *in vitro* and *in vivo* models. It lowered serum LDL-C, TG, and TC levels while increasing HDL-C in hyperlipidemic mice. Mechanistically, polydatin downregulated PCSK9 and upregulated LDLR, leading to improved lipid metabolism and suppressed tumor progression. PCSK9 knockdown enhanced these effects, while PCSK9 overexpression reversed them. Thus, polydatin exerts its anti-TNBC and lipid-lowering effects through inhibition of PCSK9 and restoration of LDLR activity.
Li et al. (2025)	Breast cancer cell lines used include MDA-MB-231-GFP (parental TNBC), 4–11 (metastatic subline with higher proliferation), MDA-MB-468 and 468-Clover (for PCSK9 overexpression), 4–3 and 4–5 (additional metastatic sublines), and non-TNBC lines UACC-893, SK-BR-3, and MCF7 (for comparison). 293T cells were used for general transfection. Female Nude and NOD/SCID mice (6–8 weeks old) were employed for lung colony and orthotopic tumor models, respectively.	PCSK9 knockdown/overexpression as the primary approach, followed by the use of inhibitors (Erlotinib, Dasatinib, Trametinib, T-5224) and chemical agents (MβCD, EGF) to validate the mechanistic pathways activated by PCSK9.	PCSK9 ↑ tumor growth (including size and weight), ↑ metastasis, and proliferation. IHC (immunohistochemistry) confirmed EGFR/HER3 activation, demonstrating that PCSK9 promotes TNBC aggressiveness *in vivo*. PCSK9 targeting LDLR internalization reduces lipid rafts in the plasma membrane, leading to EGFR-HER3 dimerization and activation of downstream pathways critical for tumor aggressiveness.
Scalambra et al. (2025)	Female mice bearing HER2^+^ breast tumors (MamBo89HER2stable model) and human HER2^+^ breast cancer cell lines (BT-474, BT-474 C5 resistant clone)	Active immunization using virus-like particle (cVLP) vaccines: cVLP-HER2 alone, cVLP-PCSK9 alone, or combined cVLP-HER2 + cVLP-PCSK9; antibody assays and serum PCSK9 measurement	Combined vaccination (cVLP-HER2 + cVLP-PCSK9) achieved complete and durable tumor regression (100% mice tumor-free >4 months), prevented relapse, and enhanced efficacy against trastuzumab-resistant cells. The synergistic effect was linked to anti-PCSK9-mediated MHC-I preservation and improved immune activation.

**TABLE 3:** Summary of experimental studies on PCSK9 and breast cancer with LDLR-independent mechanisms.

Author (Year)	Population/Model	Intervention	Main findings
Mei et al. (2025)	Mouse models (WT, Pcsk9^−/−^ knockout, and hPCSK9V474I/V474I knockin); breast cancer cell lines (EO771, MDA-MB-231)	Genetic manipulation of host PCSK9 (knockout or V474I knockin); tumor LDLR and LRP1 depletion *via* shRNA/CRISPR; recombinant PCSK9 and Evolocumab treatment	PCSK9 promotes metastasis through LRP1 degradation in tumor cells. LDLR was also degraded by PCSK9 but did not mediate metastasis, confirming LRP1 as the key causal receptor for PCSK9-driven metastatic progression.
Zhai et al. (2025)	*In vitro*: TNBC cells (MDA-MB-231), 3T3-L1 adipocytes, and tumor-associated adipocytes (CAAs). *In vivo*: High-fat diet (HFD) mouse model mimicking obesity-driven TNBC metastasis.	Manipulation of PCSK9 signaling and exosomal transfer between TNBC cells and adipocytes. • PCSK9-deficient exosome treatment. • G3BP1 knockdown to assess PCSK9 mRNA stability. • Co-immunoprecipitation to validate PCSK9–rG4 binding. • Measurement of inflammatory and matrix-remodeling markers (IL-6, IL-1β, TNF-α, MMP11)	PCSK9 acts as a molecular bridge linking lipid metabolism and immune modulation in TNBC. • TNBC-derived exosomal PCSK9 reprograms adipocytes into pro-tumor phenotypes, enhancing lipolysis and inflammation. • Loss of PCSK9 in exosomes reduces inflammatory cytokines and metastatic signaling. • G3BP1 stabilizes PCSK9 mRNA *via* rG4 binding; its inhibition downregulates PCSK9. → Highlights PCSK9 as a therapeutic target in obesity-associated TNBC progression.

#### 3.3.1 Mechanisms involving LDLR

The earliest experimental study we found was conducted by Momtazi-Borojeni et al. in 2019 (Momtazi-Borojeni et al., 2019b). By that time, PCSK9 inhibition was known to reduce lipid levels in cardiovascular disease (Bao et al., 2024), however, its effects in non-cardiovascular conditions remain unclear. Therefore, they started the investigations by developing a nanoliposome vaccine, called the anti-PCSK9 vaccine (L-IFPTA^+^), which was administered at 2-week intervals 4 times (Momtazi-Borojeni et al., 2019b). Then, they inoculated female BALB/c mice bearing 4T1 mouse breast tumor cells subcutaneously in the control and vaccine groups (Momtazi-Borojeni et al., 2019b). The results showed that PCSK9 vaccination effectively inhibited PCSK9 levels and reduced tumor growth (Momtazi-Borojeni et al., 2019b). Additionally, they reported that the vaccine group achieved an extension in survival time (Momtazi-Borojeni et al., 2019b). Subsequently, they evaluated the effect of the vaccine on the PCSK9-LDLR interaction *ex vivo*. Plasma from the vaccine-treated group showed a 46% reduction in PCSK9 binding to LDLR compared with the control group (Momtazi-Borojeni et al., 2019b). Collectively, their finding indicated that PCSK9 inhibition through vaccination can suppress the progression of breast cancer and increase the overall survival rate. Moreover, they reported a potential correlation between the effect of the vaccine and reduction of tumorigenic activity through modulation of the PCSK9-LDLR interaction (Momtazi-Borojeni et al., 2019b).

Six years later, a similar study using the PCSK9 vaccination approach was conducted. In 2025, Sclambra et al. used female mice bearing HER2^+^ breast tumors (MamBo89HER2 stable model) and human HER2^+^ breast cancer cell lines (BT-474 and its trastuzumab-resistant clone BT-474 C5) to evaluate the effects of active immunization with virus-like particle (cVLP) vaccines (Scalambra et al., 2025). The experimental groups received either cVLP-HER2 alone, cVLP-PCSK9 alone, or a combination of cVLP-HER2 and cVLP-PCSK9, followed by antibody assays and serum PCSK9 measurements (Scalambra et al., 2025). Notably, the combined vaccination (cVLP-HER2 + cVLP-PCSK9) induced complete and sustained tumor regression, with 100% of mice remaining tumor-free for more than 4 months (Scalambra et al., 2025). It also effectively prevented tumor relapse and enhanced therapeutic efficacy against trastuzumab-resistant cells. In their study, the synergistic antitumor effect was suggested to involve anti-PCSK9–mediated preservation of MHC-I expression and enhanced immune activation (Scalambra et al., 2025). Moreover, partial inhibition of PCSK9 induced by vaccination may invove the LDLR blockade in lymphocytes, thereby promoting T-cell receptor (TCR) recycling and activating CD8^+^ effector T cells (Scalambra et al., 2025).

Moving beyond vaccine-based approaches, Abdelwahed et al. identified Pseurotin A, a class of fungal secondary metabolites, as a small-molecule natural inhibitor of PCSK9 (Abdelwahed et al., 2020). Pseurotin A was found to inhibit both the secretion of PCSK9 and its interaction with LDLR (Abdelwahed et al., 2020). The authors focused on hormone-dependent breast cancer, given the increasing evidence linking lipid metabolism to cancer progression (Abdelwahed et al., 2020). To investigate the effects of Pseurotin A on PCSK9 secretion, they utilized the BT-474 (HER2+/ER+) and T47D (ER+) cell lines (Abdelwahed et al., 2020). They also established an *in vivo* orthotopic mouse model by implanting cancer cells into the mammary fat pad of mice that were fed a high-fat diet (HFD) to induce hypercholesterolemia (Abdelwahed et al., 2020). *In vivo* studies indicated that treatment with Pseurotin A significantly reduced tumor growth and local recurrence (Abdelwahed et al., 2020). Overall, the study demonstrated that Pseurotin A has a dual inhibitory function that suppresses PCSK9 secretion and disrupts the binding between PCSK9 and LDLR. In their study, the binding disruption was confirmed through molecular docking and Surface Plasmon Resonance (SPR) analyses (Abdelwahed et al., 2020).

Similar to the previous studies employing the inhibitory effect approach, Liu et al. demonstrated that Polydatin, a natural polyphenol and resveratrol derivative, acts as a PCSK9 inhibitor (Liu, 2024). In their *in vivo* experiments, the authors used the 4T1 mouse breast tumor model to represent TNBC. Mice bearing 4T1 tumors, under both normal and high-fat diet (HFD) conditions, were treated with Polydatin (Liu, 2024). For the *in vitro* experiments, human TNBC MDA-MB-231 cells and 4T1 cells were co-cultured with 3T3-L1 adipocytes to mimic the tumor–adipocyte microenvironment (Liu, 2024). The results revealed that Polydatin significantly inhibited TNBC cell proliferation, invasion, and lung metastasis in both *in vitro* and *in vivo* settings. In hyperlipidemic mice, Polydatin treatment reduced serum levels of LDL-C, TG, and TC, while increasing HDL-C (Liu, 2024). Mechanistically, Polydatin downregulated PCSK9 expression and upregulated LDLR, thereby improving lipid metabolism and suppressing tumor progression (Liu, 2024). The inhibitory effects of Polydatin on TNBC cell proliferation, invasion, and metastasis, as well as its beneficial impact on lipid metabolism, were further enhanced by PCSK9 knockdown (Liu, 2024). In contrast, PCSK9 overexpression reversed these effects (Liu, 2024). Collectively, the findings indicate that the anti-TNBC and lipid-lowering effects of Polydatin are largely mediated through the inhibition of PCSK9 and the restoration of LDLR activity.

Next, the most recent study by Li et al. (2025) utilized various breast cancer cell lines, including the MDA-MB-231-GFP line and its more aggressive metastatic variant, 4–11. Other notable lines included MDA-MB-468 and its variant, 468-Clover, which overexpresses PCSK9. Additional sublines, four to three and four to five, were also used to reflect metastatic behavior (Li et al., 2025). Female Nude and NOD/SCID mice, aged 6–8 weeks, were utilized as models to study lung colony formation and the growth of tumors in their natural environment. The experimental approach focused on altering PCSK9 levels through knockdown and overexpression techniques, followed by testing various inhibitors (Erlotinib, Dasatinib, Trametinib, T-5224) and other chemical agents (like MβCD and EGF) to explore the molecular pathways influenced by PCSK9 (Li et al., 2025). The findings revealed that increased levels of PCSK9 led to significant tumor growth, metastasis, and cell proliferation, highlighted by larger tumor size and weight (Li et al., 2025). Immunohistochemistry results showed activation of Epidermal Growth Factor Receptor (EGFR)/HER3, suggesting that PCSK9 plays a role in making TNBC more aggressive in a living organism (Li et al., 2025). That the effect of PCSK9 on LDLR internalization was found to reduce lipid raft content in cell membranes, which resulted the dimerization of EGFR and HER3, activating important signaling pathways that promote tumor growth.

#### 3.3.2 LDLR-independent mechanisms

Although many studies have suggested the roles of PCKS9 in breast cancer involving LDLR, we found two studies that give a new perspective. First, Mei et al., who provided evidence that PCSK9 plays an important role in breast cancer metastasis through mechanisms not involving LDLR. In their study, they used various mouse models, including *Pcsk9* ^−/−^ knockout and hPCSK9 gain-of-function knock-in strains, alongside breast cancer cell lines like EO771 and MDA-MB-231 (Mei et al., 2025). Their findings indicate that PCSK9 promotes tumor growth by degrading lipoprotein receptor-related protein 1 (LRP1) (Mei et al., 2025). Although PCSK9 also degrades LDLR, the absence of LRP1 affected metastasis, marking LRP1 as a key player in the way PCSK9 drives cancer progression through a distinct pathway beyond LDLR.

Adding the idea about LDLR-independent mechanism, Zhai et al. Uncovered a new pathway that PCSK9 aids tumor growth through metabolic and inflammatory interactions between TNBC and adipocytes (Zhai et al., 2025). They set up *in vitro* co-culture experiments with MDA-MB-231 cells and both regular and cancer-associated adipocytes, along with an animal model that mimics obesity-driven metastasis by using an HFD (Zhai et al., 2025). Their study revealed that exosomes from TNBC cells transport PCSK9 to adipocytes, triggering changes that promote tumor-friendly environment. This reprogramming increases lipolysis, inflammation, and changes in the extracellular matrix by activating various inflammatory markers such as IL-6, IL-1β, TNF-α, and MMP11 (Zhai et al., 2025). Additionally, they found that G3BP1 helps stabilize PCSK9 mRNA through a process involving rG4 binding, ensuring that its expression and role in metastatic signaling continue (Zhai et al., 2025). Together, the studies by Mei et al. and Zhai et al. highlight alternative pathways—beyond to LDLR—through which PCSK9 contributes to breast cancer progression.

### 3.4 Clinical relevance

#### 3.4.1 Mendelian Randomization clinical studies on PCSK9 and breast cancer

In total, we identified 12 clinical studies, including 4 Mendelian Randomization (MR) studies (Table 4). Mendelian Randomization uses genetic variants (SNPs) as proxies for specific exposures—such as genetically reduced LDL-C levels or drug inhibition effects on gene targets—to evaluate causal relationships with disease outcomes, such as cancer risk. This approach provides valuable insights into how changes in PCSK9 and lipid metabolism may impact cancer outcomes. An exploratory quantitative synthesis of the included MR studies (n = 4) reveals a consistent and significant protective effect of genetic PCSK9 inhibition on breast cancer risk. These studies, which utilize large GWAS datasets from the BCAC (n = 228,951 participants), report odds ratios (OR) for breast cancer risk reduction typically ranging from 0.81 to 0.92. Specifically, Wang W. et al. (2024) reported an OR of 0.81–0.99, and Zhang and Daxin (2024) identified an OR of 0.916. In contrast, Liang et al. (2024) found that genetically elevated LDL-C *via* PCSK9 was associated with a significantly increased risk (OR = 1.107–1.20). Collectively, these findings reinforce the causal link between the PCSK9–LDLR axis and breast cancer susceptibility, suggesting that pharmacologic PCSK9 inhibition could serve as a viable preventive strategy. From four Mendelian randomization studies, we found that they were conducted mainly on populations of European descent, drawing on the Breast Cancer Association Consortium. To note, these four studies consistently point to a single conclusion: genetically inhibiting PCSK9 appears to lower breast cancer risk significantly, and it is related to LDL-C as well.

**TABLE 4:** Summary of Mendelian randomization clinical studies on PCSK9 and breast cancer.

Author (Year)	Population/Model	Intervention	Main findings
Wang Y. et al. (2024)	Data derived entirely from public GWAS datasets (OpenGWAS) with no new biological subjects: • Population: European ancestry individuals. • Exposure datasets: LDL-C levels from two large cohorts (n = 201,678 and n = 440,546). • Outcome datasets: 14 malignant tumor types (breast, lung, gastric, hepatic, oral/pharyngeal, cervical *in situ*, bladder, thyroid, pancreatic, colorectal, renal, brain, and esophageal cancers). • Positive control: Coronary heart disease (CHD).	Drug-target Mendelian Randomization (MR) analysis simulating PCSK9 inhibitor effects through genetic proxies. • Selected 16 SNPs near PCSK9 loci (±100 kb) significantly associated with LDL-C reduction (p < 5 × 10^−8^) as instrumental variables. • Methods: Two-sample MR, using IVW, MR-Egger, weighted median, and mode-based estimates. • Sensitivity analyses: heterogeneity, horizontal pleiotropy, and reverse MR to exclude reverse causality.	Divergent causal associations between PCSK9 inhibition and cancer risk: • Protective effects: ↓ risk of breast cancer (OR 0.81–0.99, p = 2.25 × 10^−2^) and lung cancer (OR 0.65–0.94, p = 2.55 × 10^−3^). • Adverse effects: ↑ risk of gastric (OR 1.14–1.75, p = 1.88 × 10^−2^), hepatic (OR 1.46–2.53, p = 1.16 × 10^−2^), oral/pharyngeal (OR 4.49–6.33, p = 3.36 × 10^−4^), and cervical *in situ* (OR 4.56–7.12, p = 6.91 × 10^−3^) cancers. • No causal link found for bladder, thyroid, pancreatic, colorectal, renal, brain, or esophageal cancers. • Validation: Positive control confirmed—PCSK9 inhibition lowers CHD risk (OR 0.45, p = 6.17 × 10^−4^). → Overall, PCSK9 inhibitors may exert cancer-type–specific effects, protective in some tumors but detrimental in others.
Zhang and Daxin (2024)	Large-scale GWAS and eQTL datasets from individuals of European ancestry. • Breast Cancer Association Consortium (BCAC): 122,977 BC cases and 105,974 controls; subtypes ER + BC (69,501 cases) and ER−BC (21,468 cases). • Exposure data: Six lipid traits (LDL-C, HDL-C, TC, TG, APOA-I, APOB). • Validation control: CAD data from CARDIoGRAMplusC4D (60,801 cases, 123,504 controls).	Two-sample Mendelian Randomization (MR) and Summary Data-Based Mendelian Randomization (SMR) analyses to test causal links between lipid-lowering drug targets and BC risk. • Drug target genes analyzed: PCSK9, HMGCR, NPC1L1, CETP, APOB, and LDLR. • PCSK9 analysis: 9 SNPs within ±100 kb of PCSK9 locus used as genetic proxies simulating 1 mg/dL LDL reduction. • Methods: IVW (primary), MR-Egger, and SMR validation using gene expression data.	Genetic inhibition of PCSK9 was causally associated with lower breast cancer risk. • Overall BC: OR = 0.916, FDR = 0.026, indicating a protective effect of PCSK9 inhibition. • Subtype analysis: No significant causal link for ER + BC or ER−BC specifically. • Other lipid targets: HMGCR and NPC1L1 inhibition also reduced BC risk, while CETP inhibition increased it (OR = 1.194; FDR = 0.026). • Lipid traits: Higher LDL-C, HDL-C, TC, and APOA-I increased BC risk; higher TG decreased risk. → Findings reinforce that genetic PCSK9 suppression (mimicking PCSK9 inhibitors) may protect against breast cancer through lipid-lowering and possibly LDLR-mediated pathways.
Nowak and Ärnlöv (2018)	Large-scale GWAS data from European ancestry populations. • GLGC (Global Lipid Genetics Consortium): up to 188,577 individuals for plasma lipid traits. • BCAC (Breast Cancer Association Consortium): 122,977 BC cases and 105,974 controls, including ER+ and ER− subtypes.	Two-sample Mendelian Randomization (MR) using genetic instruments for lipid levels and drug target genes. • Lipid traits: LDL-C, HDL-C, TG. • Drug target MR simulated inhibition of PCSK9, HMGCR, NPC1L1, CETP, and LDLR using locus-specific SNPs. • Methods: IVW, MR-Egger, weighted median, and MR-PRESSO (to exclude pleiotropic variants).	Genetic evidence supports a causal link between lipid levels and BC risk. • Higher genetically predicted LDL-C increased overall BC risk (OR 1.09, p = 0.020), particularly ER+ BC (OR 1.14, p = 0.004). • Higher HDL-C also increased ER+ BC risk (OR 1.13, p = 0.037). • CETP variants raising HDL-C associated with higher BC risk (OR 1.07, p = 0.001). • PCSK9 inhibition (modeled genetically) was protective, significantly lowering BC risk (p = 0.014). → Findings suggest that PCSK9 inhibitors may reduce breast cancer risk through LDL-lowering mechanisms, highlighting the PCSK9–LDLR axis as a potential preventive target.
Liang et al. (2024)	GWAS datasets: 173,082 participants for LDL-C (IEU OpenGWAS) and 228,951 participants for breast cancer (122,977 BC cases, 105,974 controls; BCAC consortium, mainly European women)	Two-sample Mendelian Randomization (MR) using SNPs in PCSK9 and HMGCR loci as genetic instruments to simulate LDL-C lowering effects; main analysis by Inverse Variance Weighting (IVW)	Genetically elevated LDL-C *via* PCSK9 was causally associated with increased breast cancer risk (OR = 1.107–1.20, p < 0.01). Findings suggest that PCSK9-mediated LDL-C regulation may promote BC progression through lipid metabolic pathways, highlighting PCSK9 as a potential lipid-linked oncogenic factor.

Wang et al. identified a causal relationship between PCSK9 inhibition and breast cancer risk, suggesting a protective role of PCSK9 in breast cancer. Similar observations have been reported in lung cancer, whereas the opposite effect appears to occur in other malignancies, including gastric and liver cancers, highlighting the context-dependent role of PCSK9 across different cancer types (Wang W. et al., 2024). In another study by Zhang and Daxin, a two-sample Mendelian randomization approach demonstrated that inhibiting PCSK9 reduced the overall breast cancer risk (Zhang and Daxin, 2024). This suggests that the benefits of PCSK9 suppression might be related to its role in the LDLR pathway in managing lipid levels. Next, Nowak and Ärnlöv also employed a two-sample MR method, showing that genetic inhibition of PCSK9 led to a significant decrease in breast cancer risk (Nowak and Ärnlöv, 2018).

The aforementioned support the idea of how PCSK9 inhibitors could provide protective benefits by lowering LDL-C. Therefore, Nowak and Ärnlöv also examined the causal relationship between higher LDL-C levels and breast cancer risk. Further studies showed how increased genetically predicted LDL-C levels correlated with a higher overall risk of breast cancer, particularly in ^+^ER- positive breast cancer (Nowak and Ärnlöv, 2018). One year later, a similar study by Liang et al. used genetic markers for PCSK9 and HMGCR to assess the causal role of LDL-C, concluding that elevated LDL-C levels driven by PCSK9 were indeed associated with a higher risk of breast cancer (Liang et al., 2024).

#### 3.4.2 Non-Mendelian randomization clinical studies on PCSK9 and breast cancer

Unlike MR studies, which focus on causal relationships, non- MR clinical studies focus on direct measurements—such as circulating plasma PCSK9 levels, gene expression in tumor tissue, genetic variants, and outcomes of pharmacological interventions. We first explain two studies investigating plasma PCSK9 levels in breast cancer patients, which reported divergent results. First, Wong Chong et al. reported that plasma PCSK9 levels were significantly elevated in women with stage III breast cancer compared with those with benign breast tumors, with a positive correlation between PCSK9 concentration and disease severity (Wong Chong et al., 2022). While Ruscica et al. found that circulating PCSK9 was not a prognostic biomarker for breast neoplasm recurrence or new cancer development in a high-risk prevention trial with a median follow-up of 17 years, the longest follow-up study to date for PCSK9 observation (Ruscica et al., 2024).

Moving forward to studies using the dataset analysis, Ungvari et al. Reported a comprehensive pan-cancer analysis of PCSK9 expression and overall survival using TCGA RNA-seq and GEO microarray datasets. In breast cancer, they found that PCSK9 is significantly upregulated in invasive carcinoma compared to normal breast tissue (p = 8.16e-25). Conversely, high PCSK9 expression was associated with improved overall survival in the RNA-seq cohort (HR = 0.50, p = 0.00025). Subtype-specific analysis showed the strongest survival benefit in Luminal A (HR = 0.36, p = 0.00028) and Luminal B (HR = 0.24, p = 0.00065) tumors, showing a possible subtype-dependent protective effect. However, in the gene array cohort, no significant association was seen in any subtype, underscoring that these results are not fully consistent across database platforms and should be interpreted with caution (Ungvari et al., 2025b).

A similar finding was reported by Yi et al., who identified PCSK9 as one of eight endoplasmic reticulum stress (ERS)-related hub genes in breast cancer through TCGA and GEO transcriptomic analyses (Yi et al., 2024). Interesting yet contradictory findings highlighted. On one hand, PCSK9 was significantly overexpressed in breast cancer tumor tissue compared to normal breast tissue (p < 0.001), meaning it is upregulated in the tumor context. On the other hand, in Kaplan–Meier survival analysis, high PCSK9 expression was associated with better overall survival (HR = 0.68, 95% CI 0.49–0.93, log-rank p = 0.018), placing PCSK9 as a “favorable prognosis” gene alongside ELOVL2, IFNG, MAP2K6, MZB1, and PCSK6 in this study. Additionally, PCSK9 showed significant positive correlations with both the immune and stromal scores, suggesting it may coexist with a more immune-active tumor microenvironment. It is worth noting that the favorable prognostic association of PCSK9 reported by Yi et al. reflects the entire breast cancer population, without stratification by clinical subtype (Luminal A/B, HER2-positive, TNBC).

In the study by Wu et al. (2025), PCSK9 expression was examined in tumor tissue. They found that, in HER2^+^ breast cancer, enhanced PCSK9 expression is related to poorer prognosis, higher tumor grade, and reduced immune infiltration, particularly decreased γδ T-cell populations. In contrast, lower PCSK9 levels were associated with better neoadjuvant response and more active immune mechanisms (Wu et al., 2025). Consistently, Antipenko et al. (2025) reported that the prognostic relevance of cholesterol uptake genes, including PCSK9, varies among molecular subtypes, with HER2^+^ tumors exhibiting the highest expression (Antipenko et al., 2025).

Beyond expression studies, Mei et al. (2025) analyzed the PCSK9 germline variant rs562556 (V474I). They found that this gain-of-function mutation was associated with a markedly worse prognosis and an increased risk of distant metastasis, as it enhances PCSK9-mediated degradation of LRP1, thereby promoting metastatic progression (Mei et al., 2025). On the therapeutic side, Wei et al. (2025) have done a meta-analysis of 37 randomized controlled trials involving 108,430 participants, showing that treatment with PCSK9 inhibitors was significantly associated with a lower overall risk of neoplasms, including breast neoplasms, potentially independent of LDL-C reduction, with additional subgroup analyses suggesting possible sex-specific effects (Wei et al., 2025).

It is worth noting that across the studies of non-Mendelian Randomization clinical studies reviewed (n = 8) (Table 5), PCSK9 expression was consistently higher in cancer tissues than in normal tissues. Most studies also found links between elevated PCSK9 expression and markers of disease severity, tumor advancement, poorer prognosis, or less favorable clinical outcomes, reinforcing the proposed idea that PCSK9 may contribute to breast cancer progression. However, the association between PCSK9 expression and overall survival is somehow unclear. Although most studies found prognostic significance for PCSK9, the evidence was not entirely consistent. Two studies reported conflicting results regarding PCSK9 and survival outcomes, while one found no significant prognostic relationship. These differences may be sourced from variations in cancer types, molecular subtypes, patient populations, analytical methods, or study designs. Thus, while current research generally supports a role for PCSK9 in cancer progression, its prognostic value appears to vary across different cohorts and cancer subtypes, suggesting that its clinical significance may depend on specific contexts.

**TABLE 5:** Summary of non-Mendelian randomization clinical studies on PCSK9 and breast cancer.

Author (Year)	Population/Model	Intervention	Main findings
Wong Chong et al. (2022)	Women with breast disease: benign lesions (n = 14), stage 0 breast cancer (n = 9), and stage III breast cancer (n = 23)	Measurement of circulating plasma levels of PCSK9, ANGPTL3 and lipoprotein(a) (Lp(a)); comparison between groups	PCSK9 levels were significantly higher in women with stage III breast cancer compared to women with benign lesions (95.9 ± 27.1 ng/mL vs. 78.5 ± 19.3 ng/mL; p < 0.05). PCSK9 levels positively correlated with breast disease severity (Rho = 0.34, p < 0.05). No significant association for ANGPTL3 or Lp(a) with disease status.
Yi et al. (2024)	RNA-seq data of breast cancer patients from TCGA and validation datasets from GEO; protein expression data from Human Protein Atlas (HPA); experimental validation using 14 breast cancer and 5 normal tissue samples for qPCR.	Bioinformatic and statistical analyses to identify ER stress (ERS)-related genes linked to prognosis and immune microenvironment. • Intersection of ERS-related genes (from GeneCards) with BC DEGs → 178 genes. • LASSO and Cox regression identified 8 hub genes (ELOVL2, IFNG, IGF2BP1, MAP2K6, MZB1, PCSK6, PCSK9, POP1). • Prognostic risk model construction and subtype classification (n = 1109 BC tumors). • Immune infiltration and drug sensitivity analyses; qPCR validation for IFNG and IGF2BP1.	Identified 8 ERS-related hub genes as diagnostic and prognostic markers. PCSK9 was overexpressed in breast cancer and positively correlated with favorable prognosis (protective coefficient −0.3826). PCSK9 also showed positive correlation with immune and stromal scores, suggesting an immunoregulatory role. The 8-gene risk model achieved AUC >0.65 for OS prediction (2–6 years) and stratified BC into two molecular subtypes with distinct survival outcomes. ERS genes, including PCSK9, were mainly enriched in pathways such as PD-L1/PD-1 checkpoint signaling and choline metabolism in cancer, linking ER stress to tumor immunity and metabolism.
Antipenko et al. (2025)	Breast cancer patients from TCGA, categorized into molecular subtypes (Basal-like/TNBC, Luminal A, Luminal B, HER2+), with validation using public single-cell sequencing data	Bioinformatic and statistical analysis (DESeq2, Fisher’s test) of LDLR, LDLRAP1, and PCSK9 expression to determine prognostic value for overall survival	Prognostic relevance of cholesterol uptake genes varied by subtype: low LDLR expression indicated poor prognosis in Basal-like (TNBC) but better outcomes in Luminal A and Luminal B; PCSK9 and LDLRAP1 showed subtype-dependent trends with no significant role in HER2+ cancers. HER2+ tumors had the highest expression of cholesterol uptake genes, confirmed by single-cell data, while high *de novo* cholesterol synthesis gene expression was linked to poor prognosis in Basal-like and Luminal A subtypes.
Ruscica et al. (2024)	235 premenopausal women at high risk of breast cancer, enrolled in a phase II, double-blind, placebo-controlled prevention trial in Milan/Vicenza, Italy. • Risk groups: (1) High Gail risk (≥1.3%, n = 54) and (2) Prior IEN/T1 diagnosis (n = 181). • Follow-up: 17 years median.	Baseline plasma PCSK9 levels measured before treatment; participants randomized to tamoxifen (5 mg/day), fenretinide (200 mg/day), combination, or placebo for 2 years. • Prognostic analysis using Cox and competing risk models to assess baseline PCSK9 association with breast neoplastic events. • Correlation analyses between PCSK9, lipids (TC, LDL-C, HDL-C, TG), and 17β-estradiol.	Circulating PCSK9 was not a prognostic biomarker for breast neoplastic recurrence or new events (HR = 1.002, 95% CI 0.999–1.005, p = 0.22). PCSK9 correlated positively with total cholesterol (r = 0.277), LDL-C (r = 0.217), triglycerides (r = 0.238), and negatively with 17β-estradiol (r = −0.294). Authors conclude that plasma PCSK9 does not predict long-term breast cancer risk, but its metabolic and hormonal associations warrant further investigation in tissue-specific and mechanistic studies.
Ungvari et al. (2025b)	Patient datasets from multiple cancer types (e.g., breast, ovarian, bladder, renal, melanoma, pancreatic) using TCGA and GEO transcriptomic data	Analysis of PCSK9 expression levels across tumors and correlation with overall survival (OS) using Cox regression and Kaplan-Meier	High PCSK9 expression was significantly associated with improved overall survival (OS) in the RNA-seq breast cancer cohort (HR = 0.50, p = 0.00025). Subtype-specific analyses demonstrated the strongest survival benefit in Luminal A (HR = 0.36) and Luminal B (HR = 0.24) tumors. Analysis of tumor *versus* normal tissue showed that PCSK9 expression was significantly upregulated in invasive breast carcinoma. However, higher PCSK9 expression was associated with worse OS in bladder, renal clear cell carcinoma, melanoma, and pancreatic cancers. No significant association between PCSK9 expression and OS was observed in colon, liver, gastric, lung, prostate, head and neck cancers, or low-grade gliomas. These findings indicate that the biological and clinical roles of PCSK9 may vary across tumor contexts and molecular subtypes.
Mei et al. (2025)	Human breast cancer cohorts (TCGA-BRCA, Nik-Zainal UK, Hartwig Medical Foundation, and Swedish BC-blood early-stage cohort)	Genotyping and survival analysis of the PCSK9 germline variant rs562556 (V474I); correlation with clinicopathological features and metastasis-free survival	The V474I variant is associated with significantly worse prognosis and increased distant metastasis risk.
Wei et al. (2025)	• Study type: Comprehensive meta-analysis of 37 randomized controlled trials (RCTs). • Total participants: 108,430 adults with hyperlipidemia or at high risk for atherosclerotic cardiovascular disease (ASCVD). • Inclusion criteria: RCTs comparing PCSK9 inhibitors (PCSK9i) *versus* placebo or non-PCSK9 lipid-lowering agents, reporting neoplasm events. • Subgroup of interest: Trials with <50% male participants (female-dominant cohorts) were analyzed separately to assess sex-specific effects.	• Main intervention: PCSK9 inhibitor therapy, including Alirocumab, Evolocumab, Bococizumab, Olpasiran, and Inclisiran. • Control group: Placebo or non-PCSK9 lipid-lowering drugs. • Statistical analyses: – Random-effects meta-analysis model to estimate Risk Ratios (RRs) and 95% Confidence Intervals (CIs). – Subgroup analyses based on sex ratio, BMI, follow-up duration, and specific PCSK9 inhibitor type. – Meta-regression to assess the correlation between LDL-C reduction and neoplasm risk.	PCSK9i was associated with significantly decreased risks of overall neoplasms in patients compared with control and the risk reduction might possibly be independent of LDL-c improvement.
Wu et al., 2025	92 HER2^+^ breast cancer patient samples (IHC) and TCGA RNA-seq cohort	Bioinformatic and immunohistochemical analysis of PCSK9 expression, including GSEA, CIBERSORT immune infiltration profiling, and correlation with clinicopathologic features and survival	High PCSK9 expression was linked to poor prognosis in HER2^+^ breast cancer, higher tumor grade, and weaker immune infiltration (notably reduced γδ T cells). Low PCSK9 expression correlated with better neoadjuvant response and immune-active pathways, suggesting PCSK9 as a potential prognostic biomarker and immunotherapeutic target.

## 4 Discussion

Based on both experimental and clinical research, the findings highlight the multifaceted and complex roles of PCSK9 in breast cancer. Suggesting that its effects may depend on the specific cancer subtype, tumor microenvironment, and individual genomic variations. Most of mechanistic studies, both *in vitro* and *in vivo* agree that PCSK9’s promotion of breast cancer progression is primarily linked to its interaction with LDLR leading to lipid dysregulation (Abdelwahed et al., 2020; Huang et al., 2025; Momtazi-Borojeni et al. (2019b); Scalambra et al., 2025). Not only mechanistic exploration, there are several studies employing a PCSK9-inhibitory approach, such as Pseurotin A (Abdelwahed et al., 2020)—which then led to the finding that suppressing PCSK9 expression and disrupting the PCSK9–LDLR interaction reduced tumor growth and recurrence. This study is consistent with all Mendelian Randomization and meta-analyses of randomized controlled trials (n = 4), which also suggest that lower levels of PCSK9 and LDL-C correlate with a lower overall risk of breast tumor development (Wang Y. et al., 2024; Zhang and Daxin, 2024; Nowak and Ärnlöv, 2018; Liang et al., 2024; Wu et al., 2025).

Deep diver into molecular mechanistic pathway, two studies suggested that PCSK9 promotes tumor growth in breast cancer through an independent LDLR mechanism, targeting LRP1 (Mei et al, 2025) and directly interacting with adipocytes (Zhai et al., 2025) leading to lipid dysregulation and inflammation. For example, based on a study using TNBC, they found that when PCSK9 is overexpressed, it increases plasma LDL-C but lowers cholesterol on cell surfaces, thereby activating signaling pathways that drive cell proliferation, migration, and metastatic behavior through degradation of LRP1, which induces the activation of the EGFR/HER3/LRP1 downstream pathway (Mei et al, 2025) This suggests that PCSK9’s influence extends beyond lipid metabolism; it also affects the function of growth factor receptors, which is particularly concerning in aggressive cancer types like TNBC.

Although LRP1 and LDLR are both members of the LDL receptor family and may be influenced by PCSK9-mediated receptor degradation, their functional roles in breast cancer progression appear to differ. LDLR is primarily associated with cholesterol uptake and lipid metabolic regulation, whereas LRP1 may function more directly as a suppressor of metastatic progression. Mei et al. showed that PCSK9 promoted degradation of both LDLR and LRP1; however, the metastatic phenotype was mainly dependent on LRP1 degradation rather than LDLR loss (Mei et al., 2025). These findings suggest that potential cross-talk between LDLR-family receptors may exist through shared PCSK9-dependent trafficking or lipid-regulatory mechanisms, but current evidence indicates that LRP1 is the key causal receptor mediating PCSK9-driven breast cancer metastasis. Further studies are needed to determine whether LDLR and LRP1 interact functionally in specific breast cancer subtypes or tumor microenvironmental contexts.

Moreover, beyond its roles in metabolism and cell proliferation, PCSK9 also influences how tumors interact with the immune system. PCSK9 inhibition enhances the ability of CD8^+^ T cells, a type of immune cell, to respond to tumors (Scalambra et al., 2025). In contrast, high levels of PCSK9 can help cancer cells evade the immune response by degrading key immune molecules (Scalambra et al., 2025). In HER2-positive breast cancer, low PCSK9 levels are associated with greater immune activity, while high levels correlate with reduced infiltration of specific immune cells (Scalambra et al., 2025). These reports suggest that PCSK9 plays a significant role in mediating immune responses and shaping the effectiveness of immunotherapy.

Next, when it come to clinical studies, the implications of PCSK9 expression for patient prognosis are reported to be divergent across different tumor types (Ungvari et al., 2025b; Yi et al., 2024; Wong Chong et al., 2022). Two independent studies converge on a paradoxical yet consistent finding: PCSK9 is overexpressed in breast cancer tumor tissue compared to normal breast tissue, yet high expression is associated with improved overall survival. Ungvari et al. demonstrated that this survival benefit was strongest in Luminal A and B tumors, with independent replication in another cohort for Luminal B specifically. Similarly, Yi et al. identified PCSK9 as a hub gene predicting favorable prognosis in an unselected breast cancer cohort, with PCSK9 expression positively correlating with both immune and stromal scores (Ungvari et al., 2025a; Yi et al., 2024).

Fundamentally, breast cancer subtypes differ in their oncogenic drivers, lipid metabolism, immune composition, and receptor signaling networks. In hormone-driven tumors such as Luminal A and B, elevated PCSK9 expression may reflect a broader lipid-metabolic and endocrine-associated phenotype and may coexist with immune or stromal features linked to more favorable survival outcomes in some transcriptomic datasets (Ungvari et al., 2025b; Yi et al., 2024). The authors themselves acknowledged this interesting yet contradictory finding—that PCSK9 is overexpressed in tumor tissue yet associated with better survival in certain subtypes—and proposed that PCSK9 may have context-dependent roles that vary across molecular subtypes, stages of tumorigenesis, and tumor microenvironmental conditions. In contrast, in HER2-positive and TNBC, PCSK9 may drive aggressive behavior through immune-modulatory and receptor-independent mechanisms. A growing body of evidence suggests molecular mechanisms by which PCSK9 drives tumorigenesis in breast cancer. Liu et al. reported that PCSK9, by associating with MHC-I and promoting its lysosomal degradation, diminishes antigen presentation and facilitates immune evasion, while its inhibition promotes CD8^+^ T cell expansion (Liu et al., 2020). In TNBC specifically, PCSK9 reduces membrane cholesterol and lipid raft content, activating EGFR/HER3 and the Src/ERK/c-Jun pathway to enhance tumor growth and metastasis (Li et al., 2025). PCSK9 also mediates metabolic crosstalk between adipocytes and TNBC cells to promote tumor progression and immune evasion (Zhai et al., 2025), and facilitates LRP1 degradation to drive a pro-metastatic gene signature (Mei et al., 2025).

Therefore, rather than acting as a universal oncogenic or protective factor, PCSK9 may function as a context-dependent regulator, with its clinical significance shaped by molecular subtype, immune infiltration, cholesterol-handling pathways, and host genetic background. Future studies should integrate PCSK9 expression with *in situ* immune profiling, lipidomic analysis, receptor signaling status, and subtype-specific clinical outcomes to fully elucidate its diverse prognostic roles across breast cancer subtypes. Beyond breast cancer, PCSK9 associated with worse survival in bladder cancer (HR = 1.81), renal clear cell carcinoma (HR = 1.74), melanoma (HR = 1.69), and pancreatic cancer (HR = 2.35), reinforcing its dual, cancer-type-specific prognostic character. The authors further discussed that PCSK9’s prognostic divergence may be partly explained by its role in modulating immune surveillance—specifically through regulation of MHC-I expression on tumor cells—as well as through lipid metabolism, aging-related pathways, and tumor microenvironment interactions that differ substantially across cancer types. Indeed, there’s an urgency for research to reveal these contradictory roles of PCSK9 across various breast cancer types and to examine its potential as a therapeutic target or a prognostic marker. While direct clinical trial evidence is currently lacking, the mechanistic link between PCSK9 and receptor tyrosine kinase activation suggests a theoretical rationale for combination therapies in aggressive breast cancer subtypes. For instance, recent studies in TNBC have demonstrated that PCSK9 promotes malignancy by depleting membrane cholesterol, which subsequently disrupts lipid rafts and triggers the activation of EGFR and HER3. These findings provide a preclinical basis for exploring the synergy between PCSK9 inhibitors and EGFR-targeted agents; however, it must be emphasized that these strategies have yet to be validated in clinical settings.”

Deepening the investigation into cancer immunotherapy is also a promising direction, as multiple studies have reported the involvement of PCSK9 in enhancing antitumor immune responses. In addition to the findings by (Scalambra et al., 2025) which showed that PCSK9 inhibition increases T-cell infiltration into tumors, a groundbreaking study by (Liu et al., 2020) had reported that PCSK9 inhibition can enhance the efficacy of immune checkpoint therapy and improve therapeutic responses when combined with PD-1 inhibition. Together, these studies highlight the potential of targeting PCSK9 not only in tumor biology but also as a strategy to potentiate immunotherapy.

Despite the success of PCSK9 inhibitors in cardiovascular medicine, repurposing these agents for oncology presents significant pharmacological challenges. Tumor penetration of currently approved monoclonal antibodies, such as evolocumab and alirocumab, is likely limited by the dense desmoplastic stroma characteristic of many breast tumors, making it difficult to achieve effective concentrations within the tumor microenvironment. Additionally, optimal dosing and delivery schedules for cancer patients remain undefined and may differ from established protocols for hyperlipidemia. The long-term safety profile of PCSK9 inhibition in oncology also requires careful evaluation, particularly regarding systemic inflammation and off-target effects in extrahepatic tissues. Addressing these hurdles is essential before integrating PCSK9-targeted therapies into personalized cancer care.

At the same time, the development of new PCSK9 inhibitors represents a promising area of research. Currently, approved inhibitors are limited to monoclonal antibodies and siRNA, while small molecules and vaccines are under development (Coppinger et al., 2022). Several synthetic small-molecule and peptide-based PCSK9 inhibitors are in clinical trials and may soon expand the list of FDA-approved agents (Coppinger et al., 2022). Moreover, natural compounds such as berberine, quercetin, resveratrol, and brazilin, which are being investigated for cardiovascular benefits, may also hold potential in cancer therapy (Adorni et al., 2020; Iqbal et al., 2023). Continued innovation in PCSK9-targeted therapies holds promise for overcoming current challenges and broadening treatment options in oncology.

As for genetic studies, future research should aim to include diverse populations beyond European ethnicities and to use specific breast tissue data to better understand PCSK9’s biological significance and effects across different groups.

Undoubtedly, while the complexity and context-dependence of PCSK9 might seem challenging, it also presents an exciting opportunity. Once we fully elucidate the molecular mechanisms underlying PCSK9-induced breast cancer progression, we can advance research to develop personalized treatment strategies designed to each patient’s unique tumor characteristics and genetic background, aligning with the future of personalized medicine in oncology.

## 5 Conclusion

**FIGURE 2:**
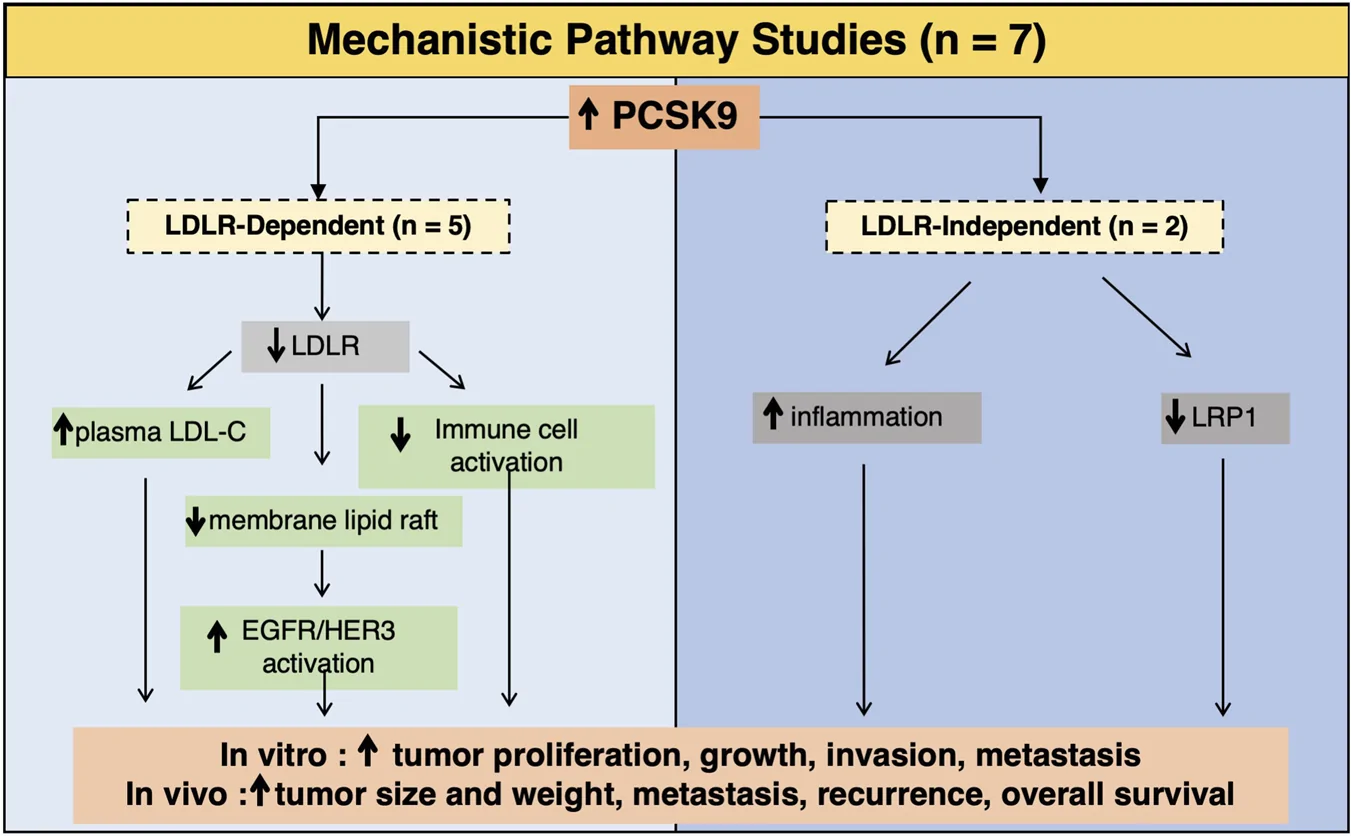
Summary of mechanistic evidence linking PCSK9 to breast cancer progression. Among the seven mechanistic studies, five reported LDLR-dependent effects of PCSK9, whereas two identified LDLR-independent mechanisms involving inflammatory pathways and LRP1 signaling. Overall, elevated PCSK9 expression was associated with enhanced tumor proliferation, growth, invasion, metastasis, recurrence, and reduced survival in both *in vitro* and *in vivo* models; PCSK9, proprotein convertase subtilisin/kexin type 9; LDLR, low-density lipoprotein receptor; LDL-C, low-density lipoprotein cholesterol; EGFR, epidermal growth factor receptor; HER3, human epidermal growth factor receptor 3; LRP1, low-density lipoprotein receptor-related protein 1.

**FIGURE 3:**
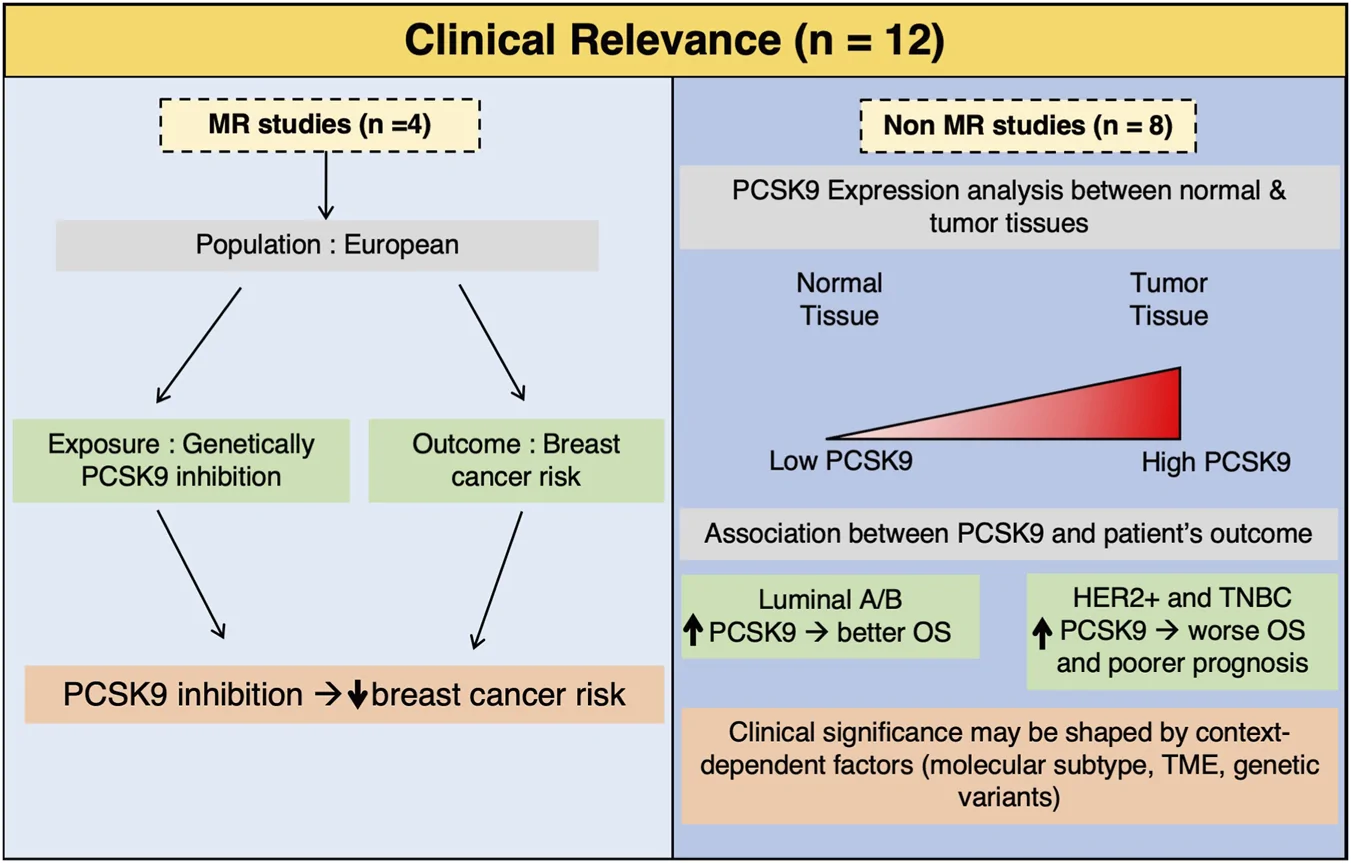
Summary of clinical evidence regarding the association of PCSK9 with breast cancer risk and prognosis. Mendelian randomization (MR) studies suggested that genetically proxied PCSK9 inhibition may reduce breast cancer risk. In non-MR clinical studies, PCSK9 expression was generally elevated in breast tumor tissues compared with normal tissues; however, its prognostic significance varied according to molecular subtype. Higher PCSK9 expression was associated with improved overall survival in luminal A/B breast cancer but poorer outcomes in HER2-positive and triple-negative breast cancer; PCSK9, proprotein convertase subtilisin/kexin type 9; MR, Mendelian randomization; HER2, human epidermal growth factor receptor 2; TNBC, triple-negative breast cancer; OS, overall survival; TME, tumor microenvironment.

In summary, this systematic review highlights the multifaceted role of PCSK9 in breast cancer, revealing that its clinical significance is context-dependent and varies across molecular subtypes. Preclinical evidence consistently demonstrates that PCSK9 promotes tumor growth and metastasis through both LDLR-Dependent pathways (e.g., activating EGFR/HER3 signaling *via* lipid-raft disruption) and LDLR-Independent pathways (notably *via* LRP1 degradation, immune evasion, and exosomal crosstalk with adipocytes), as illustrated in Figure 2. Clinically, while Mendelian Randomization studies suggest that genetic PCSK9 inhibition significantly reduces overall breast cancer risk, observational findings show divergent prognostic values (Figure 3). Elevated PCSK9 expression is associated with poorer prognosis in aggressive HER2+ and TNBC subtypes, yet correlates with improved overall survival in Luminal B tumors, where it may reflect a specific lipid-metabolic or endocrine-associated state. Although PCSK9 represents a promising therapeutic target, several limitations and challenges remain for clinical translation. Repurposing existing cardiovascular inhibitors for oncology will require addressing tumor penetration issues, establishing oncology-specific dosing, and evaluating potential toxicities within the complex tumor microenvironment. Furthermore, current genetic evidence is primarily limited to European ancestry, underscoring the need for more diverse cohort studies. Future research should validate these subtype-specific roles through spatial immune profiling and clinical trials to explore PCSK9 inhibitors as a personalized therapeutic strategy for breast cancer patients.

## Data Availability

The original contributions presented in the study are included in the article/Supplementary Material, further inquiries can be directed to the corresponding author.
