# Supplementary material for: Role of PCSK9 in breast cancer: a systematic review of its mechanistic pathways and clinical relevance
Source: Front Pharmacol. 2026 Aug 13;17:1849027. doi: 10.3389/fphar.2026.1849027 (PMC13518365; doi:10.3389/fphar.2026.1849027)
Supplement: Supplementary file 1 [file Table1.docx]

**Supplementary Table S1.** List of full-text article

| **No** | **Article Title** | | | **Include or Exclude** |
| --- | --- | --- | --- | --- |
|  | **NCBI** | **Scopus** | **Google Scholar** |  |
| 1 | PCSK9 Promotes the Malignancy of Triple‐negative Breast Cancer Cells by Reducing Cholesterol Levels at the Plasma Membrane to Activate EGFR and HER3 | PCSK9 Promotes the Malignancy of Triple-negative Breast Cancer Cells by Reducing Cholesterol Levels at the Plasma Membrane to Activate EGFR and HER3 | [PCSK9 Promotes the Malignancy of Triple‐negative Breast Cancer Cells by Reducing Cholesterol Levels at the Plasma Membrane to Activate EGFR and HER3](https://advanced.onlinelibrary.wiley.com/doi/abs/10.1002/advs.202408514) | Include |
| 2 | A Commonly Inherited Human PCSK9 Germline Variant Drives Breast Cancer Metastasis via LRP1 Receptor | A commonly inherited human PCSK9 germline variant drives breast cancer metastasis via LRP1 receptor | [A commonly inherited human PCSK9 germline variant drives breast cancer metastasis via LRP1 receptor](https://www.cell.com/cell/abstract/S0092-8674(24)01326-6) | Include |
| 3 | PCSK9 Expression and Cancer Survival : A Prognostic Biomarker at the Intersection of Oncology and Nueroscience | PCSK9 expression and cancer survival: a prognostic biomarker at the intersection of oncology and geroscience | [PCSK9 expression and cancer survival: a prognostic biomarker at the intersection of oncology and geroscience](https://link.springer.com/article/10.1007/s11357-025-01733-3) | Include |
| 4 | Circulating Levels of PCSK9, ANGPTL3 and Lp(a) in stage III Bresat Cancers | Circulating levels of PCSK9, ANGPTL3 and Lp(a) in stage III breast cancers | [Circulating levels of PCSK9, ANGPTL3 and Lp (a) in stage III breast cancers](https://link.springer.com/article/10.1186/s12885-022-10120-6) | Include |
| 5 | Polydatin Ameliorates Low-Density Lipoprotein Cholesterol and Lipid Metabolism by Downregulating Proprotein Convertase Subtisilin /Kexin Type 9 (PCSK9) in Triple-Negative Breast Cancer with Hyperlipidemia | - | Polydatin ameliorates low-density lipoprotein cholesterol and lipid metabolism by downregulating proprotein convertase subtilisin/kexin type 9 (PCSK9) in triple-negative breast cancer with hyperlipidemia | Include |
| 6 | Pseurotin A as a Novel suppressor of Hormone Dependent Breast Cancer Progression and Recurrence by Inhibiting PCSK9 Secretion and Interaction with LDL Receptor | Pseurotin A as a novel suppressor of hormone dependent breast cancer progression and recurrence by inhibiting PCSK9 secretion and interaction with LDL receptor | [Pseurotin A as a novel suppressor of hormone dependent breast cancer progression and recurrence by inhibiting PCSK9 secretion and interaction with LDL receptor](https://www.sciencedirect.com/science/article/pii/S1043661820311555) | Include |
| 7 | Effects of Immunization Against PCSK9 in an Experimental Model of Breast Cancer | Effects of immunization against PCSK9 in an experimental model of breast cancer | [Effects of immunization against PCSK9 in an experimental model of breast cancer](https://www.termedia.pl/Journal/-19/pdf-36484-10?filename=Effects%20of%20immunization.pdf) | Include |
| 8 | A Comprehensive Review on PCSK9 as Mechanistic Target Approach in Cancer Therapy | A Comprehensive Review on PCSK9 as Mechanistic Target Approach in Cancer Therapy | A Comprehensive Review on PCSK9 as Mechanistic Target Approach in Cancer Therapy | Exclude : review |
| 9 | Integrative Multi Omics Analyses Unravel the Immunological Implication and Prognostic Significance of CXCL12 in Breast Cancer | Integrative Multi Omics Analyses Unravel the Immunological Implication and Prognostic Significance of CXCL12 in Breast Cancer | [Integrative multi-omics analyses unravel the immunological implication and prognostic significance of CXCL12 in breast cancer](https://www.frontiersin.org/journals/immunology/articles/10.3389/fimmu.2023.1188351/full) | Exclude : wrong protein target |
| 10 | Immunology Update : Biologics | Immunology Update: Biologics | Immunology Update: Biologics | Exclude : review |
| 11 | The Expression of the LDLR, LDLRAP1, and PCSK9 Genes has Prognostic Significance in Triple-Negative Breast Cancer | The Expression of the LDLR, LDLRAP1, and PCSK9 Genes has Prognostic Significance in Triple-negative Breast Cancer | [The Expression of the LDLR, LDLRAP1, and PCSK9 Genes has Prognostic Significance in Triple-negative Breast Cancer](https://www.benthamdirect.com/content/journals/cmc/10.2174/0109298673291217241219055416) | Include |
| 12 | Associations of Genetically Proxied Inhibition of HMG-CoA Reductase, NPC1L1, and PCSK9 with Breast Cancer and Prostate Cancer | Associations of genetically proxied inhibition of HMG-CoA reductase, NPC1L1, and PCSK9 with breast cancer and prostate cancer (2022) | [Associations of genetically proxied inhibition of HMG-CoA reductase, NPC1L1, and PCSK9 with breast cancer and prostate cancer](https://link.springer.com/article/10.1186/s13058-022-01508-0) | Exclude : wrong focus cancer |
| 13 | Lipoprotein(a) in Patients with Breast Cancer After Chemotherapy : Exploring Potential Strategies for Cardioprotection | Lipoprotein(a) in patients with breast cancer after chemotherapy: exploring potential strategies for cardioprotection | Lipoprotein(a) in patients with breast cancer after chemotherapy: exploring potential strategies for cardioprotection | Exclude : review |
| 14 | Antitumor Activity and Molecular Mechanism of Proprotein COnvertase Subtilisin/Kexin Type 9 (PCSK9) Inhibition | Antitumor activity and molecular mechanism of proprotein convertase subtilisin/kexin type 9 (PCSK9) inhibition | Antitumor activity and molecular mechanism of proprotein convertase subtilisin/kexin type 9 (PCSK9) inhibition | Exclude : review |
| 15 | The Casual Relationship between PCSK9 Inhibitors and Malignant Tumors : A Mendelian Randomization Study Based on Drug Targeting | The Causal Relationship between PCSK9 Inhibitors and Malignant Tumors: A Mendelian Randomization Study Based on Drug Targeting | [The causal relationship between PCSK9 inhibitors and malignant tumors: a mendelian randomization study based on drug targeting](https://www.mdpi.com/2073-4425/15/1/132) | Include |
| 16 | Identification of Breast Cancer Subtypes Based on Endoplasmic Reticulum Stress-Related Genes and Analysis of Prognosis and Immune Microenvironment in Breast Cancer Patients | Identification of Breast Cancer Subtypes Based on Endoplasmic Reticulum Stress-Related Genes and Analysis of Prognosis and Immune Microenvironment in Breast Cancer Patients | Identification of Breast Cancer Subtypes Based on Endoplasmic Reticulum Stress-Related Genes and Analysis of Prognosis and Immune Microenvironment in Breast Cancer Patients | Include |
| 17 | Prognostic Value of PCSK9 Levels in Premenopausal Women at Risk of Breast Cancer-Evidence from a 17-Year Follow-Up Study | Prognostic Value of PCSK9 Levels in Premenopausal Women at Risk of Breast Cancer—Evidence from a 17-Year Follow-Up Study | [Prognostic Value of PCSK9 Levels in Premenopausal Women at Risk of Breast Cancer—Evidence from a 17-Year Follow-Up Study](https://www.mdpi.com/2072-6694/16/7/1411) | Include |
| 18 | Circulating Lipids, Lipid-Lowering Drug Targets, and Breast Cancer Risk : Comprehensive Evidence from Mendelian Randomization and Summary Data-Based Mendelian Randomization | Circulating lipids, lipid-lowering drug targets, and breast cancer risk: Comprehensive evidence from Mendelian randomization and summary data-based Mendelian randomization | [Circulating lipids, lipid-lowering drug targets, and breast cancer risk: Comprehensive evidence from Mendelian randomization and summary data-based Mendelian](https://link.springer.com/article/10.1007/s10552-024-01857-5) | Include |
| 19 | Correction : Circulating Levels of PCSK9, ANGPTL3, and 1p (a) in Stage III Breast Cancers | [Correction: Circulating levels of PCSK9, ANGPTL3 and lp (a) in stage III breast cancers](https://pmc.ncbi.nlm.nih.gov/articles/PMC11566306/) | [Correction: Circulating levels of PCSK9, ANGPTL3 and lp (a) in stage III breast cancers](https://pmc.ncbi.nlm.nih.gov/articles/PMC11566306/) | Include |
| 20 | PCSK9 V474I Germline Variant Drives Breast Cancer Metastasis | PCSK9 V474I germline variant drives breast cancer metastasis  10.1093/lifemeta/loae041 | [PCSK9 V474I germline variant drives breast cancer metastasis](https://academic.oup.com/lifemeta/article-abstract/4/1/loae041/7942712) | Exclude: review |
| 21 | Association of Serum Anti-PCSK9 Antibody Levels with Favorable Postoperative Prognosis in Esophageal Cancer | Association of Serum Anti-PCSK9 Antibody Levels with Favorable Postoperative Prognosis in Esophageal Cancer | [Association of serum anti-PCSK9 antibody levels with favorable postoperative prognosis in esophageal cancer](https://www.frontiersin.org/articles/10.3389/fonc.2021.708039/full) | Exclude : wrong focus on cancer |
| 22 | A Mendelian Randomization Study of the Effects of Blood Lipids on Breast Cancer Risk | A Mendelian randomization study of the effects of blood lipids on breast cancer risk | A Mendelian randomization study of the effects of blood lipids on breast cancer risk | Include |
| 23 | Casual Association Between Lipid-Lowering Drugs and Cancers : A Drug Target Mendelian Randomization Study | Causal association between lipid-lowering drugs and cancers: A drug target Mendelian randomization study | Causal association between lipid-lowering drugs and cancers: A drug target Mendelian randomization study | Exclude : wrong focus on cancer |
| 24 | Metabolic Dysregulation in Obese Women and the Carcinogenesis of Gynecological Tumors : A Review | Metabolic dysregulation in obese women and the carcinogenesis of gynecological tumors: A review | Metabolic dysregulation in obese women and the carcinogenesis of gynecological tumors: A review | Exclude : review |
| 25 | PCSK9 and Potential Decreased Neoplasms, Especially in Females : A Meta-Analysis | PCSK9 Inhibitor and Potential Decreased Risk of Neoplasms, Especially in Females: A Meta-Analysis | PCSK9 Inhibitor and Potential Decreased Risk of Neoplasms, Especially in Females: A Meta-Analysis | Include |
| 26 | Targeting PCSK9, Through and Innovative cVLP-based Vaccine, Enhanced the Therapeutic Activity of a cVLP-HER2 Vaccine in a PReclinical Model of HER2-Positive Mammary Carcinoma | Targeting PCSK9, through an innovative cVLP-based vaccine, enhanced the therapeutic activity of a cVLP-HER2 vaccine in a preclinical model of HER2-positive mammary carcinoma | [Targeting PCSK9, through an innovative cVLP-based vaccine, enhanced the therapeutic activity of a cVLP-HER2 vaccine in a preclinical model of HER2-positiv](https://link.springer.com/article/10.1186/s12967-025-06126-w) | Include |
| 27 | Small Interfering RNA Targeting Proprotein Convertase Subtisilin/Kexin Type 9 Might Promote Fatty Liver Disease and Hepatocellular Carcinoma Through Upregulation of CD36 | Small-interfering RNA targeting proprotein convertase subtilisin/kexin type 9 might promote fatty liver disease and hepatocellular carcinoma through upregulation of CD36 | Small-interfering RNA targeting proprotein convertase subtilisin/kexin type 9 might promote fatty liver disease and hepatocellular carcinoma through upregulation of CD36 | Exclude : commentary |
| 28 | Protein-Protein Interactions Uncover Candidate ‘Core Genes’ Within Omnigenic Disease Networks | Protein-Protein interactions uncover candidate core genes' within omnigenic disease networks | Protein-Protein interactions uncover candidate core genes' within omnigenic disease networks | Exluce : wrong Sstudy design |
| 29 |  | Prognostic implications of PCSK9 expression in HER2-positive breast cancer | [Prognostic implications of PCSK9 expression in HER2-positive breast cancer](https://www.degruyterbrill.com/document/doi/10.1515/oncologie-2024-0542/html) | Include |
| 30 |  | Osteoclast precursor cell-derived Flot2 is a novel therapeutic target for bone loss diseases |  | Exclude : Wrong protein target |
| 31 |  | Proteogenomic and observational evidence implicate ANGPTL4 as a potential therapeutic target for colorectal cancer prevention |  | Exclude : wrong focus on cancer |
| 32 |  | Risk Factors and Prevention of Cancer and CVDs: A Chicken and Egg Situation |  | Exclude : review |
| 33 |  | Mendelian Randomized Study of Protective Effect of Statins on Breast Cancer |  | Exclude : wrong focus on cancer |
| 34 |  | Comprehensive Mendelian randomization analysis of low-density lipoprotein cholesterol and multiple cancers |  | Include |
| 35 |  | [Mayo Clinic Tapestry Study: A Large-Scale Decentralized Whole Exome Sequencing Study for Clinical Practice, Research Discovery, and Genomic Educatio](https://www.scopus.com/pages/publications/85210033193?origin=resultslist)n |  | Exclude : wrong study design |
| 36 |  | Pseurotin A Validation as a Metastatic Castration-Resistant Prostate Cancer Recurrence-Suppressing Lead via PCSK9-LDLR Axis Modulation | Pseurotin A Validation as a Metastatic Castration-Resistant Prostate Cancer Recurrence-Suppressing Lead via PCSK9-LDLR Axis Modulation | Exclude : wrong focus on cancer |
| 37 |  | Towards Developing Novel Prostate Cancer Recurrence Suppressors: Acute Toxicity of Pseurotin A, an Orally Active PCSK9 Axis-Targeting Small-Molecule in Swiss Albino Mice |  | Exclude : wrong focus on cancer |
| 38 |  | PCSK9 and cancer: Rethinking the link |  | Exclude : review |
| 39 |  | Synthesis, Cytotoxicity Evaluation, and Molecular Docking Studies of Novel Pyrrole Derivatives of Khellin and Visnagin via One-Pot Condensation Reaction with Curcumin |  | Exclude : wrong study design |
| 40 |  | [Resistance and intolerance to statin](https://www.scopus.com/pages/publications/84908505976?origin=resultslist)s |  | Exclude : wrong study design |
| 41 |  | Proprotein convertases: "Master switches" in the regulation of tumor growth and progression |  | Exclude : review |
| 42 |  | Trastuzumab Cardiotoxicity: Mechanism and Management |  | Exclude : review |
| 43 |  |  | PCSK9-Mediated Metabolic Interactions between Adipocytes and Triple-Negative Breast Cancer Cells Enhance Tumor Progression and Immune Evasion | Include |
| 44 |  |  | [Anti-tumor activity of a nanoliposomal anti-PCSK9 vaccine in BALB/c mice bearing 4T1 breast cancer](https://academic.oup.com/eurheartj/article-abstract/40/Supplement_1/ehz745.0123/5594751) | Include |
| 45 |  |  | [PCSK9 inhibition: Not just LDL-Cholesterol knock down: A glimmer for cancer](https://academic.oup.com/eurheartj/article-abstract/42/12/1130/6135349) | Exclude : commentary |
| 46 |  |  | [The role of CAP1 in the progression of atherosclerosis and breast cancer](https://s-space.snu.ac.kr/handle/10371/175820) | Exclude : abstract only |
| 47 |  |  | PCSK9 inhibitor evolocumab to increase anticancer activities and reduce cardiotoxicity during doxorubicin and trastuzumab, as sequential treatment, through MyD88/NF-kB/mTORC1 pathways | Exclude : abstract only |
| total article | 28 | 41 | 36 |  |

**Supplementary Table S2.** Risk of bias assessment result using SYRCLE for *in vivo* studies

| **Author, Year** | **Sequence Generation** | **Baseline Characteristics** | **Allocation Concealment** | **Random Housing** | **Blinding of Investigators** | **Random Outcome Assessment** | **Blinding of Outcome Assessment** | **Incomplete Outcome Data** | **Selective Reporting** | **Other Bias** | **Overall ROB** |
| --- | --- | --- | --- | --- | --- | --- | --- | --- | --- | --- | --- |
| **Abdelwahed et al., 2020** | U | L | U | U | U | U | U | L | L | U | **Unclear** |
| **Li et al., 2025** | U | L | U | U | U | U | U | L | L | U | **Unclear** |
| **Liu & Zhang, 2024** | U | L | U | U | U | U | U | L | L | U | **Unclear** |
| **Mei et al., 2025** | L | L | U | U | U | U | U | L | L | U | **Low–Moderate** |
| **Momtazi-Borojeni et al., 2019** | L | L | U | U | U | U | U | L | L | U | **Low–Moderate** |
| **Scalambra et al., 2025** | U | L | U | U | U | U | U | L | L | U | **Unclear** |
| **Zhai et al., 2025** | U | L | U | U | U | U | U | L | L | U | **Unclear** |

Abbreviations: ROB, Risk of Bias; L = Low Risk of Bias; U = Unclear Risk of Bias; H = High Risk of Bias

**Supplementary Table S3.** Risk of bias assessment result using ToxRTool for *in vitro* studies

| **Author, Year** | **Cell Model Characterization** | **Exposure Assessment** | **Outcome Assessment** | **Replication & Controls** | **Selective Reporting** | **Other Bias** | **Overall ROB** |
| --- | --- | --- | --- | --- | --- | --- | --- |
| Abdelwahed et al., 2020 | Low | Low | Low | Low | Low | Moderate | Low |
| Li et al., 2025 | Low | Low | Low | Low | Low | Low | Low |
| Liu & Zhang, 2024 | Low | Low | Low | Moderate | Low | Moderate | Low–Moderate |
| Mei et al., 2025 | Low | Low | Low | Low | Low | Low | Low |
| Scalambra et al., 2025 | Low | Low | Low | Moderate | Low | Moderate | Low–Moderate |
| Zhai et al., 2025 | Low | Low | Low | Moderate | Low | Moderate | Low–Moderate |

Abbreviations: ROB, Risk of Bias.

**Supplementary Table S4.** Risk of bias assessment of included Mendelian Randomization studies using ROB-MR tool/framework.

| **Author (Year)** | **Relevance** | **Independence** | **Exclusion Restriction** | **Population Stratification** | **Overall ROB** |
| --- | --- | --- | --- | --- | --- |
| Liang et al., 2024 | Moderate | Some concerns | Moderate | Some concerns | Moderate |
| Nowak et al., 2018 | Low | Low | Some concerns | Some concerns | Low–Moderate |
| Wang et al., 2024 | Low | Low | Low | Some concerns | Low–Moderate |
| Zhang et al., 2024 | Low | Low | Low | Some concerns | Low |

Abbreviations: ROB, Risk of Bias; MR, Mendelian Randomization.

**Supplementary Table S5.** Risk of Bias Assessment of Included Non-MR Studies Using the QUIPS Tool

| **Author, Year** | **Study Participation** | **Study Attrition** | **Prognostic Factor Measurement** | **Outcome Measurement** | **Study Confounding** | **Statistical Analysis & Reporting** | **Overall ROB** |
| --- | --- | --- | --- | --- | --- | --- | --- |
| Antipenko et al., 2025 | Moderate | Low | Low | Low | Moderate | Moderate | Moderate |
| **Mei et al., 2025** | Moderate | Low | Low | Low | Moderate | Low | **Moderate** |
| Ruscica et al., 2024 | Moderate | Low | Low | Low | Low–Moderate | Low | Low–Moderate |
| Ungvari et al., 2025 | Moderate | Low | Low | Low | Moderate | Moderate | Moderate |
| Wei et al., 2025 | Low | Low | Low | Low | Low | Low | Low |
| Wong Chong et al., 2022 | Moderate | Low | Low | Moderate | Moderate | Moderate | Moderate |
| Wu et al., 2025 | Moderate | Low | Low | Low | Moderate | Low | Moderate |
| Yi et al., 2024 | Moderate | Low | Low | Moderate | Moderate | Moderate | Moderate |

Abbreviations: ROB, Risk of Bias; QUIPS, Quality in Prognosis Studies.
